# Retained Barium in the Appendix Is Difficult to Distinguish from Surgical Remnants following Laparoscopic Surgery

**DOI:** 10.1155/2018/2589080

**Published:** 2018-06-26

**Authors:** Masakazu Sato, Minako Koizumi, Takahiro Hino, Yu Takahashi, Natsuki Nagashima, Nao Itaoka, Chiharu Ueshima, Maki Nakata, Yoko Hasumi

**Affiliations:** ^1^Department of Obstetrics and Gynaecology, Mitsui Memorial Hospital, Chiyoda-ku, Tokyo, Japan; ^2^Department of Obstetrics and Gynaecology, Graduate School of Medicine, The University of Tokyo, Bunkyo-ku, Tokyo, Japan

## Abstract

Surgical materials, such as gauze, can be accidentally left inside of patients following surgery. This iatrogenic complication should be avoided and is often prevented by routine X-ray analysis after surgical abdominal procedures. We report a case of retained barium in the appendix that was difficult to distinguish from surgical remnants. A 41-year-old Japanese female was diagnosed with uterine leiomyoma and underwent laparoscopic myomectomy. The postoperative X-ray test showed a cord-like material in the lower right abdomen that was not captured in the preoperative X-ray test two months prior to the operation. Because of this difference, the area was reexamined laparoscopically. After examination, we concluded that the cord-like material in X-ray tests was in fact retained barium in the appendix. Barium can be retained in the appendix for long periods of time, and retained barium in the appendix can be captured radiographically and can mimic the appearance of surgical remnants, appearing as a cord-like material. The knowledge above combined with detailed interviews before surgery could prevent such confusion during interpretation of X-ray tests after surgery.

## 1. Introduction

It is important not to leave surgical materials (such as gauze) behind in the body after operations, as foreign bodies can cause a number of complications [1–6].

Laparoscopic surgery is now common in the field of gynaecologic operations [7]. The incision size for laparoscopic procedures is usually small, and the possibility of leaving surgical materials in the abdomen is therefore quite low [7]. However, in the present case, we could not deny the possibility and therefore had no choice but to reperform laparoscopy.

## 2. Case Presentation

**Figure 1:**
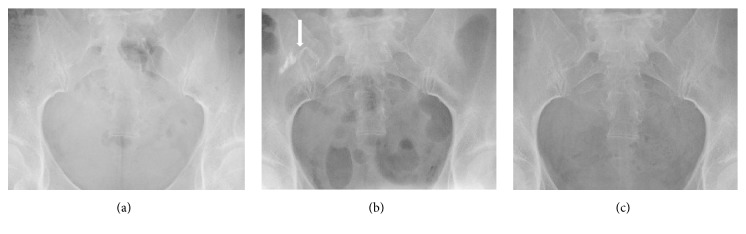
Abdominal X-ray test. (a) Preoperative X-ray test. Findings were within normal limits. (b) Postoperative X-ray test. A cord-like material was captured in the lower right abdomen. (c) X-ray test one month after operation. The cord-like material disappeared.

A 41-year-old Japanese female with no major history of past illness including surgery was diagnosed with uterine leiomyoma and underwent laparoscopic myomectomy. The operation itself was completed with no major trouble. However, the routine postsurgical abdominal X-ray test showed a cord-like material in the lower right abdomen that was not captured in the preoperative X-ray test performed two months before the operation (Figure 1). We therefore decided to investigate this finding by relaparoscopy.

**Figure 2:**
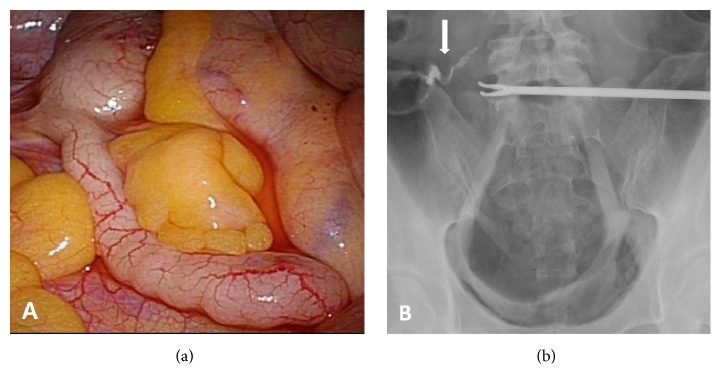
Examination by relaparoscopy. (a) Perioperative photo. No remnant was found where the cord-like material was assumed to exist. (b) Perioperative X-ray test. The shape and position of the material were found to be coincident with the appendix in (a).

During relaparoscopy, we could not find any retained material, and we performed the X-ray test again using a laparoscopic forceps as a mark (Figure 2). The cord-like material was still captured, with a minor posture change. The shape and position were found to overlap with the appendix (Figure 2), leading us to conclude that the material captured in the X-ray tests was likely retained barium in the appendix. Indeed, a postoperative interview had revealed that the patient had a barium test four weeks prior to the operation during a periodical health examination in her company.

## 3. Discussion

During the operation, we used gauze, forceps, needles, and a morcellator. Of these materials, gauze was believed to be the most likely to have been left behind from the radiographic appearance [8, 9]. We used gauze for attaching hyaluronate and carboxymethylcellulose (H-CMC; Seprafilm®) to the myomectomy site. However, the size of the incisions for laparoscopy was small (12 mm at maximum), and the possibility of leaving surgical material in the intra-abdomen was believed to be very low [7, 10]. After counting, no instruments were found to be missing or broken. Even so, we could not be 100% confident that nothing was left behind, and we had no choice but to reperform laparoscopy.

Moreover, although it is known that most patients evacuate barium within 72 hours, some patients have retained barium for a long period of time [11]. In most cases, the barium is asymptomatic, and the exact frequency of retention is unknown [11]. However, some patients present with barium appendix, in which barium can be retained and accumulated in the appendix [12–16]. In the present case, the patient had undergone a barium test four weeks prior to the operation, as determined by a postoperative interview. Indeed, the X-ray test four weeks after the operation confirmed the disappearance of the cord-like material (Figure 1).

The knowledge of the presence of retained barium (in the appendix) for a long period of time would have prevented confusion during the interpretation of an X-ray test after surgery. For this reason, the conduction of detailed interviews before surgery is extremely important.
